# Supplementary material for: CSF progranulin increases in the course of Alzheimer's disease and is associated with sTREM2, neurodegeneration and cognitive decline
Source: EMBO Mol Med. 2018 Nov 27;10(12):e9712. doi: 10.15252/emmm.201809712 (PMC6284390; doi:10.15252/emmm.201809712)
Supplement: Supplementary file 1 — Appendix [file EMMM-10-e9712-s001.pdf]

Appendix

CSF progranulin increases in the course of Alzheimer's disease and is associated with sTREM2, neurodegeneration and cognitive decline

Authors: Marc Suárez-Calvet, Anja Capell, Miguel Ángel Araque Caballero, Estrella Morenas-Rodríguez, Katrin Fellerer, Nikolai Franzmeier, Gernot Kleinberger, Erden Eren, Yuetiva Deming, Laura Piccio, Celeste M. Karch, Carlos Cruchaga, Katrina Paumier, Randall J. Bateman, Anne M. Fagan, John C. Morris, Johannes Levin, Adrian Danek, Mathias Jucker, Colin L. Masters, Martin N. Rossor, John M. Ringman, Leslie M. Shaw, John Q. Trojanowski, Michael Weiner, Michael Ewers, Christian Haass for the Dominantly Inherited Alzheimer Network and for the Alzheimer's Disease Neuroimaging Initiative.

Table of contents of the Appendix:

Appendix Tables 2
Table S1. CSF PGRN as a function of mutation status, age, gender and *APOE ε4* status in the DIAN sample 2
Table S2. Modelling the relationship between CSF PGRN and other biomarkers as a function of EYO 2
Table S3. CSF PGRN estimates (pg/ml) in MCs and NCs as a function of EYO including outliers and participants with EYO > +20 4
Table S4. Characteristics of the complete ADNI sample 5
Table S5. Summary of the statistical analysis including the CSF PGRN outliers in the ADNI sample 6
Table S6. Associations of CSF PGRN with cognitive measures including the CSF PGRN outliers 6
Table S7. Associations of CSF PGRN with cognitive measures in the SNAP category in the ADNI sample 7
Table S8. Associations of CSF PGRN with other CSF biomarkers in ADAD and late-onset AD, including AD CSF biomarkers outliers 7
Table S9. Participant inclusion criteria in the ADNI sample 7
Appendix Figures 8
Fig. S1. Comparison of CSF PGRN measurements between MSD ELISA and Adipogen ELISA 8

Appendix Tables

Table S1. CSF PGRN as a function of mutation status, age, gender and *APOE ε4* status in the DIAN sample

	Excluding outliers				Including outliers		
**Fixed effects**	**Parameter estimate (B)**	**SE**	***P*-value**		**Parameter estimate (B)**	**SE**	***P*-value**
Mutation carrier	110	26.2	0.00004*		98.6	29.1	0.0008*
Age	2.18	1.23	0.079		2.31	1.38	0.095
Female gender	-64.6	25.6	0.012*		-75.0	28.5	0.009*
APOE ε4 status	-5.78	28.6	0.840		-3.96	31.8	0.901

Summary of the linear model for CSF PGRN (outcome variable) as a function of mutation status, age, gender and *APOE* ε4 status. The DIAN sample contained 3 CSF PGRN outliers (2 NC and 1 MC), as defined as 3 standard deviations below or above the group mean. Noticeably, the analysis excluding the outliers (reported in the main text) and including the outliers yielded similar results.
*Significant differences.
Abbreviations: APOE, apolipoprotein E; SE, standard error.

Table S2. Modelling the relationship between CSF PGRN and other biomarkers as a function of EYO

In order to study the changes of CSF PGRN and other biomarkers as a function of EYO, we examined the following models:

- **1st order model (m1):** Marker ~ Mutation + Gender + EYO + EYO*Mutation [fixed effects] + Family affiliation [random effect]
- **1st and 2nd order model (m2):** Marker ~ Mutation + Gender + EYO + EYO*Mutation + EYO^2 + EYO^2*Mutation [fixed effects] + Family affiliation [random effect]
- **1st, 2nd and 3rd order model (m3):** Marker ~ Mutation + Gender + EYO + EYO*Mutation + EYO^2 + EYO^2*Mutation + EYO^3 + EYO^3*Mutation [fixed effects] + Family affiliation [random effect]

The final model for each biomarker was chosen on the basis of the Akaike information criterion (AIC), as a measure of the goodness of the fit, and we also compared each model by a likelihood-ratio test (F-test) to the immediate high-rank model. A test rendering a $P < 0.05$ denotes that adding the higher rank term significantly improves the model fit. For CSF PGRN, the first order term EYO model rendered the lower AIC and, when compared to higher rank models, the likelihood-ratio test (F-test) did not render significant results, denoting that adding the higher rank term did not improved the model fit.
The final model chosen for each biomarker is highlighted in bold characters.

	AIC	Likelihood-ratio test vs lower rank model *(P value)*
**CSF PGRN**		
**m1**	**-108.5**	**-**
m2	-107.5	0.224
m3	-103.7	0.901
**PIB-PET (precuneus)**		
m1	193.2	-
**m2**	**190.7**	**0.038**
m3	193.7	0.607
**CSF A$\beta_{1\text{-}42}$**		
**m1**	**2632.0**	**-**
m2	2635.7	0.861
m3	2634.8	0.087
**CSF T-tau**		
**m1**	**2417.9**	**-**
m2	2421.9	0.997
m3	2420.8	0.077
**CSF sTREM2**		
m1	-188.3	-
m2	-188.8	0.106
**m3**	**-193.5**	**0.013**
**FDG-PET (precuneus)**		
**m1**	**-66.6**	**-**
m2	-63.5	0.648
m3	-60.0	0.762
**MRI Hippocampal volume**		
m1	3172.6	-
**m2**	**3168.2**	**0.015**
m3	3207.5	0.777
**MMSE**		
m1	1229.4	-
**m2**	**1226.1**	**0.025**
m3	1229.0	0.591

Table S3. CSF PGRN estimates (pg/ml) in MCs and NCs as a function of EYO including outliers and participants with EYO > +20

	Estimated years from expected symptom onset (EYO)							
	**-25**	**-20**	**-15**	**-10**	**-5**	**0**	**+5**	**+10**
Non-carriers	974	978	983	988	992	997	1002	1006
Mutation carriers	992	1022	1052	1082	1112	1142	1172	1202
Difference	18	44	69	94	120	145	170	196
95% CI	[-101, 138]	[-61, 148]	[-25, 163]	[6, 182]	[31, 208]	[50, 240]	[64, 277]	[74, 317]
P-value	0.763	0.412	0.149	0.036*	0.008*	0.003*	0.002*	0.002*

Mean estimated levels of CSF PGRN were obtained by a lineal model including gender, mutation status, EYO and the interaction between mutation status and EYO as covariates (see statistics section). For each EYO, the group difference, 95% CI and the *P*-value for the two-sample independent *t* test is reported. Unlike Table 2 in the main text, we included here the 3 PGRN outliers (2 NC and 1 MC) and those participants with EYO > +20 (1 NC and 2 MC). The results including or excluding these participants are similar. Differences are calculated from unrounded values. *Significant differences.
Abbreviations: CI, confidence interval.

Table S4. Characteristics of the complete ADNI sample

	CDR = 0 (n = 306)				CDR = 0.5 (n = 605)				CDR = 1 (n = 106)			
	**A-/TN-** (n = 128)	**A+/TN-** (n = 56)	**A+/TN+** (n = 48)	**A-/TN+** (n = 74)	**A-/TN-** (n = 120)	**A+/TN-** (n = 96)	**A+/TN+** (n = 289)	**A-/TN+** (n = 100)	**A-/TN-** (n = 2)	**A+/TN-** (n = 15)	**A+/TN+** (n = 81)	**A-/TN+** (n = 8)
Age, y	72.5 (5.37)	73.2 (5.96)	76.5 (5.37)	74.6 (6.56)	70.0 (7.55)	72.7 (7.71)	73.3 (7.05)	73.3 (8.12)	89.2 (1.63)	76.3 (6.05)	73.8 (9.27)	79.9 (7.43)
Female, %	48.4	46.4	52.1	55.4	45.0	21.9	42.6	46.0	0	33.3	50.6	25.0
APOE ε4 carriers, %	14.8	39.3	58.3	21.6	20.8	50.0	76.5	33.0	0	53.3	74.1	12.5
GRN rs5848 TT carriers, %*	5.20	13.2	6.90	11.3	14.4	8.96	13.6	9.23	0	0	14.8	33.3
Education, y	16.3 (2.79)	16.5 (2.74)	16.5 (2.47)	16.4 (2.56)	16.1 (2.70)	16.2 (2.81)	15.9 (2.87)	15.9 (2.62)	17.0 (0.00)	16.2 (2.51)	15.1 (2.78)	14.9 (1.96)
**Cognitive tests scores**												
ADNI-Mem	1.14 (0.59)	1.04 (0.61)	0.86 (0.56)	1.11 (0.51)	0.65 (0.68)	0.13 (0.58)	-0.28 (0.64)	0.32 (0.67)	-0.78 (0.37)	-0.97 (0.43)	-1.02 (0.46)	-0.81 (0.32)
ADNI-EF	0.94 (0.75)	0.74 (0.70)	0.40 (0.61)	0.83 (0.64)	0.56 (0.75)	0.038 (0.81)	-0.12 (0.79)	0.44 (0.82)	-1.14 (0.41)	-1.01 (0.73)	-1.07 (0.78)	-0.49 (0.80)
ADAS-Cog11‡	5.96 (3.12)	5.85 (3.06)	6.54 (3.01)	5.31 (2.78)	7.84 (3.67)	10.6 (4.54)	13.2 (5.34)	9.00 (4.22)	17.0 (2.83)	20.6 (7.33)	23.2 (6.98)	20.1 (5.15)
ADAS-Cog13‡	8.94 (4.57)	9.27 (4.51)	10.2 (4.45)	8.40 (3.99)	12.4 (5.64)	16.9 (6.51)	21.2 (7.38)	14.5 (6.59)	27.5 (3.54)	30.5 (8.71)	34.6 (7.95)	30.0 (5.78)
MMSE	29.1 (1.12)	28.9 (1.23)	29.0 (1.20)	29.2 (1.00)	28.3 (1.61)	27.6 (2.24)	26.3 (2.39)	27.8 (2.00)	23.0 (1.41)	23.6 (2.13)	22.7 (2.06)	22.8 (1.91)
CDR-SB	0.016 (0.09)	0.054 (0.16)	0.073 (0.21)	0.034 (0.13)	1.32 (0.77)	1.64 (0.99)	2.09 (1.09)	1.50 (0.96)	6.75 (1.77)	5.50 (1.23)	5.62 (1.17)	5.81 (0.88)
**CSF biomarkers, pg/ml†**												
T-tau	184 (31.8)	166 (41.2)	329 (77.5)	324 (72.2)	185 (37.7)	171 (40.1)	382 (135)	337 (108)	234 (6.08)	188 (38.8)	395 (137)	468 (213)
P-tau_{181P}	16.3 (2.87)	15.4 (4.09)	33.0 (8.93)	29.0 (7.36)	15.9 (3.29)	15.7 (4.19)	39.1 (14.5)	31.4 (13.3)	19.3 (1.20)	17.4 (3.53)	39.4 (15.1)	41.3 (21.9)
$A\beta_{1-42}$	1455 (227)	724 (193)	712 (173)	1542 (233)	1428 (245)	640 (199)	636 (167)	1437 (291)	1330 (14.9)	538 (185)	575 (159)	1480 (269)
PGRN	1502 (279)	1394 (365)	1569 (305)	1760 (356)	1556 (348)	1463 (291)	1541 (343)	1705 (330)	1885 (490)	1704 (405)	1649 (375)	1974 (475)

Data are expressed as mean and standard deviation (SD) or percentage (%), as appropriate. **GRN rs5848* genotype was available in 650 participants (64% of the complete ADNI sample). ‡there were 2 subjects without ADAS-Cog11 score and 6 subjects without ADAS-Cog13 score. †The CSF core biomarkers measurements were performed using the electrochemiluminiscence immunoassays Elecsys Total-tau CSF, phosphor-tau(181P) CSF and β-amyloid(1-42) CSF. Abbreviations: A: amyloid-β biomarker status; $A\beta_{1-42}$: amyloid-β 42; APOE, apolipoprotein E; ADAS-Cog, Alzheimer's disease Assessment Scale – cognitive subscale; ADNI-Mem: ADNI memory composite score; ADNI-EF: ADNI executive function composite score; CDR: clinical dementia rating; CDR-SB: clinical dementia rating Sum of Boxes; CSF, cerebrospinal fluid; MMSE, Mini-Mental State Examination; N: neurodegeneration biomarker status; P-tau_{181P}, tau phosphorylated at threonine 181; T: tau pathology biomarker status; T-tau, total tau; y, years.

Table S5. CSF PGRN as a function of age, gender and *APOE ε4* status in the ADNI sample

	Excluding outliers				Including outliers		
Fixed effects	Parameter estimate (B)	SE	*P*-value		Parameter estimate (B)	SE	*P* -value
Age	0.0006	0.0004	0.118		0.0009	0.0005	0.056
Female gender	-0.031	0.006	<0.0001*		-0.034	0.007	<0.0001*
APOE ε4 status	-0.011	0.006	0.066		-0.015	0.007	0.024*

The ADNI sample contained 11 CSF PGRN outliers, as defined as 3 standard deviations below or above mean. PGRN was log-transformed to achieve a normal distribution.
*Significant differences.
Abbreviations: APOE, apolipoprotein E; SE, standard error.

Table S6. Associations of CSF PGRN with cognitive measures including the CSF PGRN outliers

	**Model 1** (unadjusted)			**Model 2** (adjusted by age, gender, *APOE* ε4 and education)			**Model 3** (also adjusted by $A\beta_{1-42}$ and T-tau)	
	β	*P*		β	*P*		β	*P*
**ADNI-Mem**	-0.151	0.001*		-0.151	0.001*		-0.082	0.050
**ADNI-EF**	-0.152	0.001*		-0.158	0.0004*		-0.114	0.010*
**ADAS-Cog 11**	+0.147	0.001*		+0.147	0.001*		+0.098	0.028*
**ADAS-Cog 13**	+0.149	0.001*		+0.152	0.001*		+0.099	0.023*
**MMSE**	-0.106	0.021*		-0.108	0.017*		-0.058	0.188
**CDR-SB**	+0.092	0.045*		+0.104	0.023*		+0.042	0.340

Associations between CSF PGRN and cognitive measures were studied in the participants of the AD *continuum* including the CSF PGRN outliers (n = 480), as defined as 3 standard deviations below or above mean. They were assessed by three different linear regression models. The standardized regression coefficients (β) and the *P*-values are shown. Note that the results including or excluding the outliers (Table 5) rendered similar results. There were 2 subjects without ADAS-Cog11 score and 6 subjects without ADAS-Cog13 score.
*Significant differences.
Abbreviations: $A\beta_{1-42}$: amyloid-β 42; ADAS-Cog, Alzheimer's disease Assessment Scale – cognitive subscale; ADNI-Mem: ADNI memory composite score; ADNI-EF: ADNI executive function composite score; CDR-SB: clinical dementia rating Sum of Boxes; MMSE, Mini-Mental State Examination; T-tau: total tau.

Table S7. Associations of CSF PGRN with cognitive measures in the SNAP category in the ADNI sample

	Model 1 (unadjusted)			Model 2 (adjusted by age, gender, *APOE* ε4 and education)			Model 3 (also adjusted by $A\beta_{1-42}$ and T-tau)	
	β	*P*		β	*P*		β	*P*
**ADNI-Mem**	-0.086	0.250		+0.040	0.561		+0.048	0.483
**ADNI-EF**	-0.023	0.757		+0.095	0.170		+0.081	0.247
**ADAS-Cog 11**	+0.066	0.375		-0.030	0.686		-0.034	0.639
**ADAS-Cog 13**	+0.085	0.253		-0.012	0.874		-0.019	0.785
**MMSE**	-0.069	0.357		+0.020	0.787		+0.036	0.628
**CDR-SB**	+0.059	0.428		-0.019	0.798		-0.022	0.765

Associations between CSF PGRN and cognitive measures were studied only in the participants of the SNAP category (n = 182) and were assessed by three different linear regression models. The standardized regression coefficients (β) and the *P*-values are shown.
Note that, unlike the results in the AD *continuum* category, CSF PGRN is not associated with cognitive impairment in any cognitive tests investigated.
Abbreviations: $A\beta_{1-42}$: amyloid-β 42; ADAS-Cog, Alzheimer's disease Assessment Scale – cognitive subscale; ADNI-Mem: ADNI memory composite score; ADNI-EF: ADNI executive function composite score; CDR-SB: clinical dementia rating Sum of Boxes; MMSE, Mini-Mental State Examination; SNAP: suspected non-Alzheimer's pathophysiology; T-tau: total tau.

Table S8. Associations of CSF PGRN with other CSF biomarkers in ADAD and late-onset AD, including the CSF biomarkers outliers

	Autosomal Dominant Alzheimer's disease (DIAN study)			Late-onset Alzheimer's disease (ADNI study)		
	**Non-carriers (n = 87)**	**Mutation carriers (n = 131)**		**Healthy controls (n = 129)**	**Continuum AD (n = 480)**	**SNAP (n = 182)**
sTREM2	β = +0.159 *P* = 0.167	β = +0.516 *P* < 0.0001*		β = +0.033 *P* = 0.723	β = +0.339 *P* < 0.0001*	β = +0.296 *P* < 0.0001*
T-tau	β = +0.200 *P* = 0.071	β = +0.282 *P* = 0.002*		β = +0.047 *P* = 0.616	β = +0.261 *P* < 0.0001*	β = +0.132 *P* = 0.080
$P\text{-tau}_{181P}$	β = +0.230 *P* = 0.038*	β = +0.185 *P* = 0.045*		β = +0.051 *P* = 0.583	β = +0.259 *P* < 0.0001*	β = +0.130 *P* = 0.082
$A\beta_{1-42}$	β = +0.186 *P* = 0.090	β = +0.149 *P* = 0.132		β = -0.016 *P* = 0.853	β = +0.063 *P* = 0.178	β = +0.194 *P* = 0.010*

The standardized regression coefficients (β) and the *P*-values are shown and were computed using a linear model adjusting for age, gender and *APOE* ε4. The $A\beta_{1-42}$ values used for the associations test are those based on an extrapolation curve since the upper technical limit is 1700pg/ml. Of note, the results excluding (Fig 6 and 7) or including the outliers rendered similar results.
*Significant differences.
Abbreviations: $A\beta_{1-42}$: amyloid-β 42; T-tau: total tau; $P\text{-tau}_{181P}$: tau phosphorylated at Threonine 181; SNAP: suspected non-Alzheimer's pathophysiology.

Table S9. Participant inclusion criteria in the ADNI sample

- Participants belonging to ADNI 1/GO/2.
- Clinical diagnosis of CN, SMC, early and late MCI, or mild AD.
- Availability of CSF samples at baseline with measured CSF $A\beta_{1-42}$ concentration.
- Availability of clinical diagnosis and neuropsychological tests (ADNI-Mem, ADNI-EF and ADAS-Cog) at baseline and at ≥ 2 yearly follow-ups.

Appendix Figures

Fig. S1. Comparison of CSF PGRN measurements between MSD ELISA and Adipogen ELISA

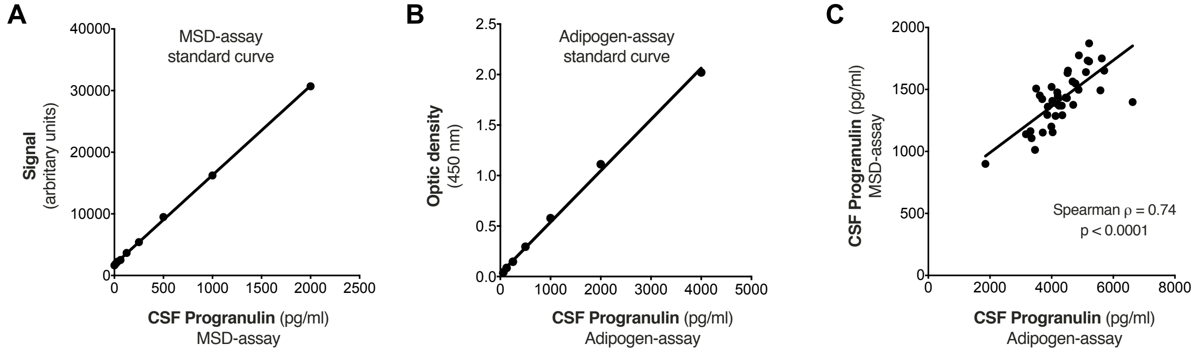

We simultaneously measured 39 CSF pool samples in the MSD and the Adipogen ELISAs and the standard curves for both ELISAs are shown (**A**, **B**). We compared the values from both ELISAs (**C**) and, although the range of the values differed, the values were highly correlated between them (Spearman $\rho = 0.74$; $P < 0.0001$).
