# Supplementary material for: CSF progranulin increases in the course of Alzheimer's disease and is associated with sTREM2, neurodegeneration and cognitive decline
Source: EMBO Mol Med. 2018 Nov 27;10(12):e9712. doi: 10.15252/emmm.201809712 (PMC6284390; doi:10.15252/emmm.201809712)
Supplement: Supplementary file 2 — Expanded View Figures PDF [file EMMM-10-e9712-s002.pdf]

# Expanded View Figures

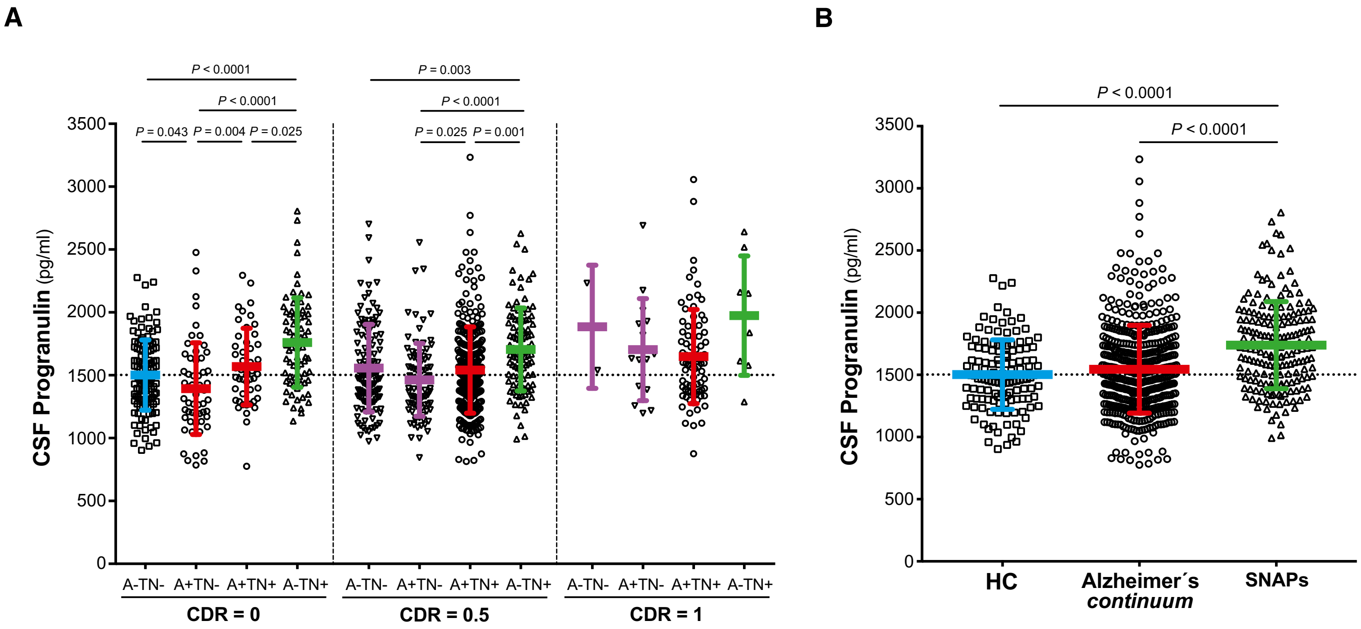


**Figure EV1. CSF PGRN levels in the A/T/N framework.**

A Scatter plot representing the levels of CSF PGRN for each of the four biomarker profiles within each clinical staging, as defined by CDR. CDR = 1 stage comprises some biomarker profiles that do not contain enough participants to perform statistical analysis but are nevertheless shown in the figure for completeness. Each biomarker category is represented in a different colour. Healthy controls are depicted in blue, Alzheimer's *continuum* category in red and SNAP category in green. Purple depicts biomarker profiles not assigned in any category in the present study.

B Scatter plot grouping the three biomarker categories: healthy controls, all the participants belonging to the Alzheimer's *continuum* category and the suspected non-Alzheimer's pathophysiology (SNAP) category.

Solid bars represent the mean and the standard deviation (SD). *P*-values were assessed by a one-way analysis of covariance adjusted for age, gender and *APOE* ε4, followed by Bonferroni corrected pair-wise *post hoc* comparisons. The analysis and graphs were performed excluding PGRN outliers (1 "healthy control", 1 "Preclinical AD A+/TN−", 4 "AD CDR = 0.5", 3 "CDR = 0.5 A−TN−", 1 "CDR = 0.5 A+TN−", 1 "AD CDR = 1"). Including them yielded a similar result.

Data information: A: amyloid-β biomarker status; AD: Alzheimer's disease; CDR: clinical dementia rating; CSF, cerebrospinal fluid; N: neurodegeneration biomarker status; SNAP: suspected non-Alzheimer's pathophysiology; T: tau pathology biomarker status.

 © 2018 The Authors

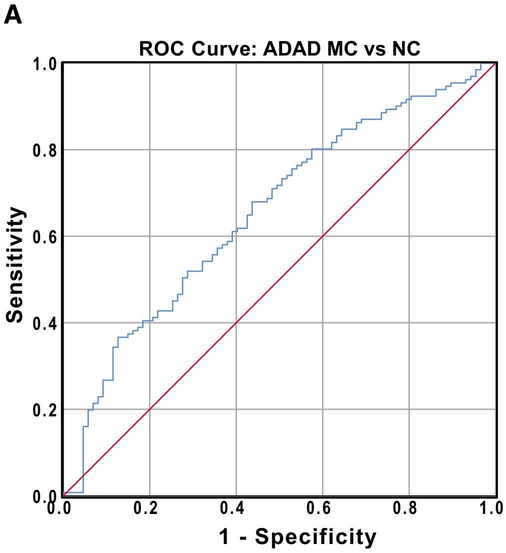


| | ADAD MC vs NC |
|---|---|
| AUC | 0.655 |
| 95% CI | 0.581 – 0.729 |
| *P*-value | 0.0001 |
| Cutoff | 940 pg/ml |
| Sensitivity | 68 % |
| Specificity | 56 % |

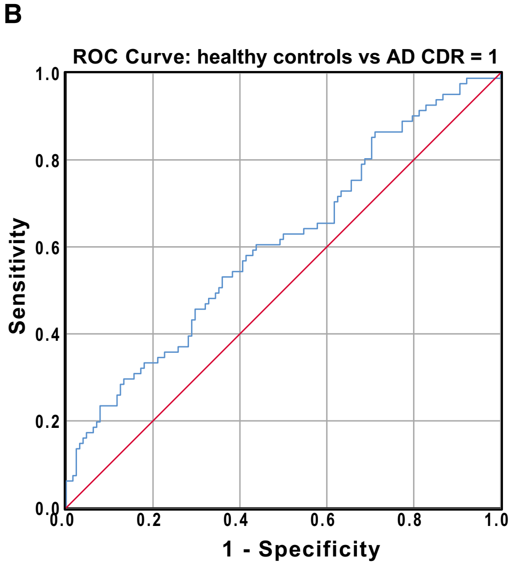


| | AD CDR = 1 vs HC |
|---|---|
| AUC | 0.607 |
| 95% CI | 0.528 – 0.686 |
| *P*-value | 0.009 |
| Cutoff | 1591 pg/ml |
| Sensitivity | 53 % |
| Specificity | 64 % |

**Figure EV2. Diagnostic accuracy of CSF PGRN as an AD biomarker.**

ROC analysis was performed to test the accuracy to discriminate between ADAD mutation carriers and non-carriers of the DIAN study (A) and late-onset AD with mild dementia (CDR = 1) from healthy controls of the ADNI study (B). The areas under the curve (AUCs), their 95% confidence interval (CI) intervals and the *P*-values are reported. Optimal cut-offs were derived based on the Youden index, and the sensitivity and specificity were calculated based on these cut-offs. AD: Alzheimer's disease; ADAD: autosomal dominant Alzheimer's disease; AUC: area under the curve; CDR: clinical dementia rating; HC: healthy controls; MC: mutation carriers; NC: non-carriers.

© 2018 The Authors
